# An Exploratory Study of the Frequency of Central Nervous System Tumors by Type in the Central Texas Military and Civilian Populations

**DOI:** 10.7759/cureus.13885

**Published:** 2021-03-14

**Authors:** Jennifer Murillo, Jonathan Artz, Ankita Brahmaroutu, Spencer Gunnell, Jared Benge, Kristopher Lyon, Ekokobe Fonkem

**Affiliations:** 1 Neurology, Baylor Scott & White Medical Center - Temple, Temple, USA; 2 Psychiatry, Baylor Scott & White Medical Center - Temple, Temple, USA; 3 Neurology, University of Pennsylvania Perelman School of Medicine, Philadelphia, USA; 4 Neuropsychology, University of Texas at Austin Dell Medical School, Austin, USA; 5 Neurosurgery, Baylor Scott & White Medical Center - Temple, Temple, USA; 6 Neurology, Barrow Neurological Institute, Phoenix, USA

**Keywords:** agent orange, brain metastases, central nervous system tumors, meningioma, military cancer

## Abstract

Background: The types of central nervous system (CNS) tumors in a patient population with a history of military service were compared to the types of CNS tumors in a similar patient population without a military service history to determine if a relationship exists between military service and CNS tumor type.

Methods: This study analyzed data for adult patients diagnosed with an intra- or extra-axial CNS tumor from January 2016 to July 2019. One cohort was constructed of patients who had a history of military service (MIL), and the other cohort was made of patients who did not have a history of military service (NMIL). Appropriate parametric and non-parametric analyses were used to compare frequencies of tumor types between cohorts adjusting for potential confounders.

Results: We identified 2001 patients (MIL, *n* = 190; NMIL, *n* = 1811). In the MIL cohort, most patients were males, younger, and more racially diverse. In the primary analysis, the MIL cohort showed higher diagnoses of metastatic tumors compared with the NMIL cohort (X^2^(1)= 3.71, *p*=.05). The MIL cohort also showed lower diagnoses of meningioma compared to the NMIL cohort. There was no statically significant difference between cohorts or tumors after adjusting for primary source by gender.

Conclusions: MIL experience was associated with lower diagnoses of meningioma but higher diagnoses of metastatic cancer, providing support that there may be potential differences in tumor types between patients with a history of military service and those without military history regarding primary CNS tumor frequency.

## Introduction

In the past decades, studies comparing the incidence rates of central nervous system (CNS) tumors between patients with a history of military service and those without have delivered conflicting results. One well-studied example involves veterans of the Persian Gulf War. The Persian Gulf War linked exposure to nerve agents released during the March 1991 weapons demolition in Khamisiyah, Iraq, with an increased risk of death from brain cancer [1]. The relative risk of brain cancer deaths in veterans exposed to two or more days of these toxins (RR=3.26; 95% CI=1.33, 7.96) increased significantly when compared to veterans exposed to only one day (RR=1.72; 95% CI=0.95, 3.10) [2-3]. In addition to nerve agents, Gulf War veterans who were exposed to oil well fire smoke had an increased risk of brain cancer mortality (RR=1.81; 95% CI=1.00, 3.00) when compared to veterans who were not exposed [2]. However, a later study about this same military population found that there was no increase in brain cancer incidence when compared to non-Gulf War veterans [4]. Other studies, such as those reviewed by the Committee to Review the Health Effects in Vietnam Veterans of Exposure to Herbicides, have demonstrated insufficient evidence to definitively link veteran exposure to Agent Orange with increased incidence of CNS tumors, despite the fact that it is a unique chemical to which the civilian population was largely not exposed [5]. Additionally, a different study evaluated active duty United States Air Force personnel from 1989-2002 and found that there was a statistically significant decrease in the incidence of brain neuroepithelial cancer in the military population compared to standardized incidence ratios in the general population [6]. 

In an Italian study, a case-control analysis performed for newly diagnosed brain tumors from 1990-1999 revealed a statistically significant association between military occupation and two of the most common brain tumors, gliomas and meningiomas [7]. Additional studies have shown a correlation between general military exposures (radiation, chemical carcinogens, etc.) and CNS tumor growth. There is some evidence that exposure to low frequency/microwave electromagnetic fields is associated with an increased risk of developing CNS tumors [6,8]. A retrospective cohort study comparing Canadian military members to the general population found that while Canadian military members who served any time between January 1, 1976, and May 31, 2015, overall had lower incidences of cancer, males were found to have a statistically significant increased risk of developing brain cancer [9]. Another study in Iowa looking at occupation and risk for histologically confirmed brain gliomas reported a statistically significant higher odds ratio for developing a brain glioma in men who had served in an unspecified role in the military [10]. 

Many prior studies had limitations that were similar in nature. In some of the studies, the statistics were underpowered due to limited number of military CNS tumor cases analyzed [5,9-11]. Study design was also a limitation, such as a hospital-based case-control design which may not accurately reflect the general population [11]. One other potential issue in some of the studies was reporting. Military personnel have the option of seeking medical care through a military healthcare system or through another healthcare system, which could underestimate the true burden of CNS tumors in military personnel [12]. Likewise, confounding factors such as relative health may have impacted the results since Gulf War veterans were slightly younger and possibly healthier compared to non-Gulf War veterans [4]. Limitations in the other studies included not having data for pre- and post-environmental and occupational exposures as well as a comprehensive list of chemicals for which military personnel may have been exposed [1]. In those with Gulf War exposure to chemicals that may have caused brain cancer, air samples were not taken and computer models were used to determine areas of exposure to sarin [2]. In some studies, family history of brain cancer was not addressed [2]. Other factors such as behavioral risk factors were not taken into account because of lack of data [2]. 

This study aims to compare the frequency of primary and secondary CNS tumor types in those with a history of military service against those without a military history in the Central Texas region. Secondly, this study aims to explore how demographic differences may relate to different patterns of nervous system tumor development in Central Texas. Bell County and the adjacent areas within Central Texas are ideal for studying our population of interest. As a state, Texas is second only to California regarding total veteran population, with the Bell County area specifically having a veteran population density of 13.5%, which is more than twice the state average [13-14]. Although we are unable to provide exact numbers, the Baylor Scott and White System serves as a primary referral site for management of CNS tumors and other serious neurological conditions for Veterans Healthcare Facilities in the region, as the Central Texas Veterans Health Care System does not provide neuro-oncology services. Baylor Scott and White is the only major medical system in the region providing comprehensive neurological and neurosurgical care with the Baylor Scott & Medical Center itself being located just one mile west of the Olin E. Teague Veterans' Medical Center in Temple, Texas.

## Materials and methods

Study design and data source

This was a retrospective cohort study using patients’ information from our electronic medical record (EMR) with specific International Classification of Diseases (ICD)-10 codes (Table 1) which were dated January 1, 2016, to July 1, 2019. We extracted these data and reviewed encounters at the Baylor Scott & White Medical Centers in Temple, TX; Waco, TX; Round Rock, TX; and Temple Cancer Center. 

**Table 1:** ICD-10 Codes for primary and secondary CNS Tumors ICD: International Classification of Diseases, CNS: central nervous system

Description	ICD 10
Neuroendocrine carcinoma	C7A.1
Leptomeningeal disease	C70.1
Cerebrum, except lobes and ventricles, includes basal ganglia, unspecified lobe of cerebral cortex, corpus striatum, globus pallidus, hypothalamus, and thalamus	C71.0
Frontal lobe	C71.1
Temporal lobe, which includes hippocampus and uncus	C71.2
Parietal lobe	C71.3
Occipital lobe	C71.4
Ventricles, which includes choroids plexus and ventricle floor	C71.5
Cerebellum, not otherwise specified, which includes cerebellopontine angle	C71.6
Brain stem, which includes cerebral peduncle, medulla oblongata, midbrain, and pons	C71.7
Other parts of brain, which includes corpus callosum and tapetum. This code also includes a malignant neoplasm of contiguous or overlapping sites of brain whose point of origin cannot be determined	C71.8
Brain unspecified and cranial fossa unspecified	C71.9
Glioma: malignant neoplasm of brain not otherwise specified	C71.9
Oligodendroglioma	C71.9
Astrocytoma (also known as glioma) includes anaplastic and glioblastoma	C71.9
Ependymoma	C71.9
Medulloblastoma	C71.6
Benign neoplasms of the brain	D33.2
Secondary brain tumors AKA metastasis/unknown origin	C79.31, C80.1
Germinoma	C80.1
Acoustic neuroma (schwannoma) — Malignant	D36.10
Acoustic neuroma (schwannoma) — Benign	D36.10
Meningioma	D32.0
CNS lymphoma	C85.89
Hemangioblastoma CNS	D18.02
Craniopharyngioma	D44.4
Ganglioglioma	D48.9
Adenoma	D35.2
Mass of brain, extra axial in left temporal region	G93.9
Neurofibromatosis	Q85.00
Neurofibromatosis I	Q85.01
Neurofibromatosis II	Q85.02
Neurofibromatosis III	Q85.09
Neoplasm of brain	D49.6
Neurocytoma	D33.2
Leptomeningeal metastases	C79.49
Schwannomatosis	Q85.03
Neoplasm of pituitary gland	D49.7
Neoplasm of uncertain behavior of brain	D43.2
Neoplasm of uncertain behavior of brainstem/infratentorial/cerebellum	D43.1
Neoplasm of uncertain behavior of brain, supratentorial	D43.2
Neoplasm of uncertain behavior of cerebral meninges	D42.0
Neoplasm of uncertain behavior of cerebral ventricle/cerebrum/frontal lobe/occipital/parietal/temporal	D43.0
Bladder cancer metastasized to brain	C67.9
Breast cancer metastasized to brain	C50.919, C50.912, C50.911
Colon cancer metastasized to brain	C18.9

Case selection

From the EMR, we identified a cohort of 110 patients ≥18 years of age, with a primary or secondary CNS nervous system tumor diagnosis who had a recorded history of military payor information and had “self” as relationship in that payor information. Patients missing information on veteran status or who did not have a defined tumor type identified via chart review were excluded. Patient demographics were obtained from the EMR data. Data collected included age, gender, and race. The records were stratified by gender for each cohort. 

Defining military group membership

Two cohorts were constructed based on military service history payor type listed at any time as Tricare, Veterans’ Administration, and/or TriWest Healthcare Alliance and also had the relationship of self were placed into the military cohort (MIL). Another cohort comprised patients who had previous payor information as listed with relationship being spouse, child, or other and all other patients extracted that did not have the previously named payor information was labeled as the non-military (NMIL) cohort. 

Statistical analysis

In this exploratory study, we examined the frequency of tumor diagnoses over the same period in veteran and civilian samples. The Student’s t-test was used to compare age between cohorts while potential confounding nonparametric variables (gender, race, and ethnicity) were compared via Chi-Squared testing. Distribution of tumor type between the NMIL and MIL cohort was compared via Chi-Squared analysis and was repeated exclusively on males. Primary source of metastatic disease was also compared via Chi-Squared testing with repeat analyses to compare metastatic disease incidence in NMIL and MIL males given the differing incidence of cancer type between sexes. All statistics were conducted using JASP (JASP Team, 2019).

## Results

In total, 2782 charts were initially reviewed. After case-by-case evaluation by the primary author, 781 charts were omitted because of diagnostic errors in the chart (i.e. non-nervous system cancer). This left a total of 2001 cases included in the current study with 190 (9.45%) being in the MIL cohort. Tumor types with less than 10 reported cases were merged into the “Other” category. 

The MIL cohort (age 55.19 years [sd=15.99]) was slightly younger than the NMIL cohort (58.74 years [sd=17.23]; t(1999)=2.723, p=.007). Further demographic descriptions of the patients are found in Table 2. The MIL cohort tended to be more racially diverse than the civilian sample and disproportionately male.

**Table 2:** Demographic and Descriptive Data from Military Service (MIL) and Non-Military Service (MIL) Cohorts

Variable		MIL (N=190)	NMIL (N=1811)	Contrast
Gender	Male (%)	86.32	37.94	X^2^(1)=164.68, p<.0001
Age	Years	55.19	58.74	t = 2.72, p < .007
Race	American Indian or Alaska Native	2.11	.28	X^2^(4)=42.25, p<.0001
	Asian (%)	1.05	1.55	
	Black or African American (%)	24.74	12.54	
	White or Caucasian (%)	60.00	77.64	
	Other/Unknown (%)	12.1	7.99	
Ethnicity	Hispanic (%)	6.32	11.82	X^2^(2)=21.39, p<.0001
	Not Hispanic (%)	85.79	85.70	
	Other/Unknown (%)	7.89	2.48	

The distribution of tumor type differed by cohort (Table 3) to a statistically significant degree (X2(16)=47.39, p<.001). When our population was analyzed as a whole, the MIL cohort had a higher proportion of metastases (X2(1)=3.71, p=.05) and a lower proportion of meningioma cases in comparison to the NMIL cohort (X2(1)=10.39, p<.01). There was no significant difference in percentage of glioblastoma multiforme (GBM) diagnoses between the cohorts.

**Table 3:** Percentage of Tumor Type by Cohort MIL: Military Service, NMIL: Non-Military Service, GBM: glioblastoma multiforme, DNET: dysembryoplastic neuroepithelial tumors

	MIL (N=190)	NMIL (N=1811)
Tumor Type	%	%
mets	33.68	27.11
pituitary adenoma	19.47	16.90
meningioma	16.32	22.64
GBM	5.79	9.39
neurofibromatosis	3.68	4.58
schwannoma	3.68	3.70
spinal cord lesion	3.16	0.66
oligodendroglioma	2.63	1.77
ependymoma	2.63	0.99
OTHER	2.11	2.82
cyst	2.11	1.71
astrocytoma	1.58	2.26
lymphoma	1.58	0.50
craniopharyngioma	1.05	0.88
DNET	0.53	0.00
unknown brain lesion	0.00	2.82
hemangioma	0.00	1.2

Given the potential confounding effect of gender, our population of 851 males was analyzed separately and maintained a statistically significant difference in overall tumor distribution (X2(16)=38.24, p=.001). Among males, there was no longer a significant difference in meningioma incidence between cohorts (X2(1)=.158, p=.691). The male NMIL cohort had a higher proportion of GBM cases in comparison to their MIL counterparts that was significant (X2(1)=3.93, p<.05). These changes are in line with the incidence rates reported for GBM and meningiomas in the 2019 Central Brain Tumor Registry of the United States (CBTRUS) Statistical Report and are discussed in further detail below [11].

Follow-up analysis of the source of metastatic disease is presented in Table 4. Overall, the distribution of metastatic source differed between the cohorts (X2(4)=11.15, p=.02), with more breast cancer in the NMIL cohort and more lung cancer in the MIL cohort. However, given the fact that primary sources of metastatic tumors differ between genders, we re-ran the analyses looking only at NMIL versus MIL males and explored differences in primary tumor type [15]. When genders were compared directly, no statistically significant differences were observed (X2(3)=3.54, p=.32).

**Table 4:** Sources of metastatic tumors in the full sample and males only MIL: Military Service, NMIL: Non-Military Service, GBM: glioblastoma multiforme, DNET: dysembryoplastic neuroepithelial tumors

	Males + Females		Males Only	
	MIL (N=190)	NMIL (N=1811)	MIL (N=164)	NMIL (N=687)
Tumor Type	%	%	%	%
Metastatic Lesions	33.68	27.11	35.98	32.17
Pituitary Adenoma	19.47	16.90	18.29	14.41
Meningioma	16.32	22.64	14.02	13.68
GBM	5.79	9.39	6.71	12.81
Neurofibromatosis	3.68	4.58	3.66	4.22
Schwannoma	3.68	3.70	4.27	2.62
Spinal Cord Lesion	3.16	0.66	3.05	0.73
Oligodendroglioma	2.63	1.77	3.05	2.91
Ependymoma	2.63	0.99	1.83	1.60
Other	2.11	2.82	1.83	4.08
Cyst	2.11	1.71	2.44	1.31
Astrocytoma	1.58	2.26	1.83	2.77
Lymphoma	1.58	0.50	1.83	0.15
Craniopharyngioma	1.05	0.88	0.61	1.46
DNET	0.53	0.00	0.61	0.00
Unknown Brain Lesion	0.00	2.82	0.00	3.79
Hemangioma	0.00	1.27	0.00	1.31

## Discussion

Because of the larger occupational hazards inherent to military service, there may be a difference between primary and secondary CNS (including intra-axial and extra-axial) tumor rates between MIL and NMIL. However, there is little comprehensive research on these tumor types in the United States military population. Although nervous system tumors are the most common form of solid tumors in children, they are less common in adult populations, being the eighth most common cancer among those greater than 40 years old [11]. As such, studies are more frequently conducted on cancers more common than primary and secondary CNS cancers. The Veteran Affairs Central Cancer Registry (VACCR) study found that the Veterans Affairs’ and United States’ male cancer population were similar compared to the United States general cancer population [15]. However, another study compared the common cancer incidence rates for the general United States population and found an increase in prostate cancer in military personnel [16]. Although this is not primary or secondary CNS cancer, it is suggestive that cancer patterns vary between military and non-military populations [17]. 

Previous studies have suggested that there is an elevated risk of meningioma and GBM development in military service populations [7,18]. Our initial analysis showed fewer meningioma diagnoses in the Central Texas MIL population over the three-year time period observed, which was eliminated when cohorts were stratified by gender as women made up a larger proportion of the NMIL cohort (62.1% vs 13.7% in men) and as a gender had double the occurrence of meningiomas (28.20% vs 13.75% in men). This is consistent with the findings of the most recent CBTRUS report showing meningiomas are more than twice as common in women compared to men [11]. 

Our evaluation does have several limitations. One such limitation is that if a veteran had a non-military payor listed, they were not placed in the MIL cohort. Those with military experience also may have been included in the NMIL cohort if this information was missing. Additionally, meningiomas can also be incidental findings and given our younger population, incidental meningiomas may not be known if they are relatively healthy with little brain imaging. This is also a limitation stratifying by payor type so that if the military payor types used for classification were not listed as payor in our system, the patient was not placed in the MIL cohort. There is selection bias in that the data is from one geographical source. Also, male gender representation outweighs female representation due to military patient demographics. Future efforts would benefit from data allowing classification by military branch, length of service, and position so that studies could also evaluate for possible risk factors based on exposure and further evaluate based on tour location, position-related exposure in given duties such as radio/microwave electric field frequency, radiation and hazardous/environmental clean-up efforts. Occupational information after completion of military service would also be valuable to further quantify additional exposures. 

Lastly, our initial comparison between the MIL and NMIL cohorts showed no difference in percentage of GBM diagnoses. When our male population was analyzed separately, the predominantly female NMIL cohort had a greater increase in proportion of GBM cases than the MIL cohort, reflective of the fact that GBMs are more than 1.5 times as common in men [11]. The lower occurrence of GBMs in our MIL cohort would appear to contradict findings from previous studies showing increased risk of CNS tumors in military personnel [7]. Given that the NMIL cohort was predominantly composed of Caucasians, in whom GBMs are two times more common than in African Americans [11], the lower occurrence of GBMs in our MIL group could be attributable to the difference in racial composition. The diversity of our MIL cohort is actually in line with the racial demographics of the United States Military Services as a whole, which have been more racially diverse than the civilian workforce since at least the 1970s. Most recent reports show 30.1% of active duty enlisted military as being non-white compared to 24.1% of their age-matched civilian counterparts. Given the differing rates of CNS tumor prevalence amongst different racial and ethnic groups [11], our study serves to highlight the importance of accounting for such factors when studying a military population. It also highlights a factor readers should be cognizant of with international studies of risk associated with military service, such as the case-control studies conducted by Italian authors Fallahi et al. whose study population was 93% Caucasian and included only 10 minority cases [7].

The metastatic source differed between the MIL and NMIL cohorts in our study. There were more breast cancer diagnoses in the NMIL cohort, and more lung cancer diagnoses in the MIL cohort. Breast and lung cancer are the most common cancers in the world, with both being the most common sources of metastases to the brain as well [19]. Because our MIL cohort was largely male and breast cancer affects vastly more women than men, our analysis was rerun on males exclusively with no significant difference between groups. Lung cancer was the most common primary cancer in both groups of males at 79.3% in the MIL group and 69.86% in NMIL group. Although not to the point of significance the differing rate does warrant further investigation for occupational risk factors or whether rates of smoking or other known risk factors differ between groups.

## Conclusions

Our study serves as a first look into the frequency of CNS tumors in the Central Texas veteran population. Although it is hindered by many of the limitations present in prior studies, it highlights some of the demographic differences present in the military population and emphasizes how impactful these differences can be. Due to the limitations of retrospective research, we were also unable to ensure which wartime periods, if any, were associated with our MIL cohort data. A next step to this research would be to explore the relationship between military exposures like radiation and possible increase in frequency of brain tumors that were previously reported. Given the fact that more than two million military personnel have been deployed to southwest Asia and exposed to numerous potential carcinogens such as exhaust from military vehicles, fumes from fires, weapons, and depleted uranium since 2001, identifying past and current carcinogenic substances that military personnel are exposed to will be vital to protecting the health of future military members. 

## References

[1] Bullman TA, Mahan CM, Kang HK, Page WF (2005). Mortality in US army Gulf War veterans exposed to 1991 Khamisiyah chemical munitions destruction. Am J Public Health.

[2] Barth SK, Kang HK, Bullman TA, Wallin MT (2009). Neurological mortality among US veterans of the Persian Gulf War: 13-year follow-up. Am J Ind Med.

[3] Barth SK, Dursa EK, Bossarte RM, Schneiderman AI (2017). Trends in brain cancer mortality among US Gulf War veterans: 21-year follow-up. Cancer Epidemiol.

[4] Young HA, Maillard JD, Levine PH, Simmens SJ, Mahan CM (2010). Investigating the risk of cancer in 1990-1991 US Gulf War veterans with the use of state cancer registry data. Ann Epidemiol.

[5] National Academies of Sciences, Engineering Engineering, and Medicine (2018). Veterans and Agent Orange: Update 11 (2018). National Academies of Sciences E and Medicine (US), Board on Population Health and Public Health Practice, National Academies of Sciences E and Medicine (US), Health and Medicine Division. Veterans and Agent Orange: Update.

[6] Yamane GK (2021). Cancer incidence in the US Air Force: 1989-2002. Aviat Space Environ Med.

[7] Fallahi P, Elia G, Foddis R, Cristaudo A, Antonelli A (2017). High risk of brain tumors in military personnel: a case control study. Clin Ter.

[8] Grayson JK (1996). Radiation exposure, socioeconomic status, and brain tumor risk in the US Air Force: a nested case-control study. Am J Epidemiol.

[9] Rolland-Harris E, Simkus K, Weeks M (2019). Burden of cancer mortality in the Canadian armed forces, 1976-2012: a retrospective cohort study. Cancer Epidemiol Biomarkers Prev.

[10] Zheng T, Cantor KP, Zhang Y, Keim S, Lynch CF (2001). Occupational risk factors for brain cancer: a population-based case-control study in Iowa. J Occup Environ Med.

[11] Ostrom QT, Cioffi G, Gittleman H, Patil N, Waite K, Kruchko C, Barnholtz-Sloan JS (2019). CBTRUS statistical report: primary brain and other central nervous system tumors diagnosed in the United States in 2012-2016. Neuro Oncol.

[12] Arrigo RT, Boakye M, Skirboll SL (2012). Patterns of care and survival for glioblastoma patients in the veterans population. J Neurooncol.

[13] (2020). United States Census Bureau QuickFacts: Texas. https://www.census.gov/quickfacts/TX.

[14] (2021). National Center for Veterans Analysis and Statistics, Texas Summary Report (FY2017). https://www.va.gov/vetdata/docs/SpecialReports/State_Summaries_Texas.pdf.

[15] Barnholtz-Sloan JS, Sloan AE, Davis FG, Vigneau FD, Lai P, Sawaya RE (2004). Incidence proportions of brain metastases in patients diagnosed (1973 to 2001) in the Metropolitan Detroit Cancer Surveillance System. J Clin Oncol.

[16] Zullig LL, Jackson GL, Dorn RA, Provenzale DT, McNeil R, Thomas CM, Kelley MJ (2012). Cancer incidence among patients of the US Veterans Affairs health care system. Mil Med.

[17] (2020). Cancer Stat Facts: Brain and Other Nervous System Cancer. https://seer.cancer.gov/statfacts/html/brain.html.

[18] Zhu K, Devesa SS, Wu H (2009). Cancer incidence in the US military population: comparison with rates from the SEER program. Cancer Epidemiol Biomarkers Prev.

[19] Nayak L, Lee EQ, Wen PY (2012). Epidemiology of brain metastases. Curr Oncol Rep.

